# Australian veterinarians' perspectives on the contribution of the veterinary workforce to the Australian animal health surveillance system

**DOI:** 10.3389/fvets.2022.840346

**Published:** 2022-08-18

**Authors:** Lynne Hayes, Jennifer Manyweathers, Yiheyis Maru, Emma Davis, Robert Woodgate, Marta Hernandez-Jover

**Affiliations:** ^1^Graham Centre for Agricultural Innovation (NSW Department of Primary Industries and Charles Sturt University), Wagga Wagga, NSW, Australia; ^2^School of Agricultural, Environmental and Veterinary Sciences, Charles Sturt University, Wagga Wagga, NSW, Australia; ^3^Commonwealth Scientific and Industrial Research Organization, Canberra, ACT, Australia; ^4^Global Veterinary Solutions Pty. Ltd, Yass, NSW, Australia

**Keywords:** emergency animal disease, preparedness, veterinarians, surveillance, capacity

## Abstract

This study investigated the involvement of private veterinarians in surveillance activities and the veterinary workforce's contribution to the Australian animal health surveillance system. The perception that there is overall a decreased engagement by veterinarians in surveillance outcomes at a time when there is increased need for bolstering of surveillance systems was investigated. Three key questions were considered: (1) What is the current contribution of private veterinarians to the Australian surveillance system? (2) What is the veterinary professions capacity to assume a more prominent role in surveillance? (3) What is the interest and ability of the veterinary profession in Australia to undertake this surveillance role now and into the future? Semi-structured telephone interviews were conducted with 17 private veterinarians with data analyzed qualitatively to identify key themes. Results demonstrate that private veterinarians are aware of their responsibilities and are engaged in surveillance activities at both formal and informal levels. The key challenges associated with current and future contributions were related to workload, remuneration, conflicts of interest and clarity over how responsibility for surveillance is shared amongst those involved in the system. The study has demonstrated that even amongst an engaged population, barriers do need to be addressed if private veterinarians are to be tasked with increasing their involvement in animal health surveillance activities.

## Introduction

In Australia, the animal health surveillance system is multifaceted, and includes active programs that target exotic and nationally significant endemic diseases that are funded by state/federal government and industry as well as passive or general surveillance activities. Jurisdictional veterinary authorities, private veterinarians, industry and non-government organizations and producers are key stakeholders carrying out animal health surveillance. Australian governments and industries through a 3-y National Animal Health Surveillance Business Plan determine current surveillance programs and priorities and their effective implementation (1). The Australian livestock industries have a strong export focus, with active surveillance programs allowing the country to meet market access requirements. These active programs target diseases such as Transmissible Spongiform Encephalopathies, economically important arboviruses (eg. Bluetongue virus, Akabane and bovine ephemeral fever), screw worm fly and avian influenza in wild birds.

General surveillance, the regular monitoring of the health of animals and reporting suspected unusual signs of disease and deaths, plays a vital role in the prompt detection of an emergency animal disease (EAD) and is critical for a rapid and effective response (2, 3). Whilst the importance of general (and active) surveillance in protecting Australia's livestock industry from both endemic, notifiable and exotic disease is irrefutable (4, 5), these activities involve a complex system reliant on the actions of producers and a host of other animal health stakeholders. While much research has focused on the producer's role in agricultural systems and their uptake of biosecurity practices, it is important to understand that producers do not operate in isolation. Producers are supported by a larger network that includes both government and private veterinarians (6–8). A key part of the veterinary role is to diagnose and investigate disease. Veterinarians are also trained and skilled in observing otherwise undetected signs of disease. Their interactions with producers also provide opportunites to hear about suspicions of abnormalities in health and welfare. As such, veterinarians are central to the upholding of animal health systems.

Both private and government veterinarians have been identified by livestock producers as trusted sources of information for animal health and as being influential on animal management practices (9, 10). In Australia, State and Territory Governments have legislative requirements for veterinarians, laboratories and livestock owners (indeed all citizens) to report suspicion or confirmation of notifiable diseases (11). There is also the opportunity for private veterinarians to engage with producers in a preventative disease advisory role, particularly pertinent given their position as trusted advisors (12, 13).

Private veterinarians' general activities, such as networking within their profession and with owners and managers of animals and providing information to stakeholders in local and regional animal health networks, also contribute to surveillance outcomes. There is, therefore, a further depth to veterinary involvement with scope to add value to field activities through broadening private veterinary practitioner's services to include active field surveillance on behalf of the government. Frawley (14), in a review of rural veterinary practices, recommended commissioning veterinarians to undertake specific surveillance activities that require strengthening in particular locations or for particular diseases.

Factors such as changes in climate, increased movement of people and animals, international trade and changes in land use patterns (15–17), have resulted in an increasing need for biosecurity vigilance. This encompasses the practices that are undertaken on an individual farm through to systems put in place by industry and government to identify and respond to disease threats (18). The importance of global transparency in the reporting of disease has perhaps never been as apparent as now, as the world navigates its way through a global pandemic. In the animal health space, the growth in big data, whilst having the potential to enhance surveillance capabilities (19), also means that surveillance data may be under greater scrutiny as access to information on shared platforms increases.

These changes in dynamics have coincided with a considered government focus of moving toward a framework of “shared responsibility” for biosecurity (20–22). In Australia, over the past 20 years, there has been a systematic rationalization of government veterinary service roles (23). It appears there has been a reduction in surveillance at a time that societal, trade and environmental factors have driven an increased need for diligence in scanning for and detecting disease. In the case of animal disease surveillance, there is an expectation that the withdrawal of government services will be taken up by an increase in activity by private veterinarians (23). One example of a government program that uses private veterinarians to boost Australia's capacity for the early detection of emerging and emergency animal diseases is the National Significant Disease Investigation (NSDI) Program. This scheme subsidizes private veterinary practitioners and state and territory laboratories to investigate significant disease incidents in livestock and wildlife.

There are currently 13,993 registered veterinarians in Australia (24). In the 2021 Australian Veterinary Association Workforce Survey (*n* = 3,456) it was reported that of the 2,762 participants working in clinical practice, just over 6% worked primarily with livestock. This is in comparison to the number working in small animal practice (57.2%) and those working in mixed animal practice with “some” large animals (17.7%) (24). This demonstrates the shift in the demographic profile of the veterinary profession with what was traditionally an agricultural profession now showing a proportional decrease in diversification of many rural veterinary businesses away from solely large animal to a mixed practice that also caters for companion animals; a move often needed to ensure financial viability given the low number of producers regularly engaging the services of veterinarian (14). Indeed, there is evidence that the time that private veterinarians spend on farm has reduced with some farmers having not had a veterinarian on farm for a number of years (22, 23, 25). This is not a situation that is unique to Australia. A survey carried out across 28 countries through the Federation of Veterinarians of Europe (FVE), reported that 78.5% of respondents described a shortage of veterinarians in rural areas. Some of the suggested reasons for this shortage were a preference for companion animal practice, economic hardship experienced by farmers, working conditions, corporatization of the veterinary profession and low demand for services (26).

In response to the retraction in government support, it appears that the veterinary profession and veterinary practitioners are not assuming the level of involvement with biosecurity and surveillance that is expected (23). In this paper, we consider surveillance as one tool that supports biosecurity legislation, and while considering the engagement with biosecurity practices generally is important, this paper focuses on the specific work of disease surveillance. While it is important to understand the barriers to engagement in biosecurity and related activities, it is equally important to understand motivation and capacity as a way of understanding potential for behavior uptake (27, 28). A number of factors are likely contributors to the disparity between expectation and reality. In addition to an awareness by private veterinarians that this is indeed part of their role, a mix of personal, professional, and business aspects of veterinary practice may impact private veterinarians' ability to further contribute to surveillance activities (29, 30). The workload of private veterinarians, particularly those in rural and regional areas (31), is of concern and has been shown to be a contributing factor to veterinarians leaving the profession (32). As such, it is important to understand the capacity of the veterinary profession to provide additional surveillance services.

This research was part of a larger study investigating the veterinary professions contribution to animal disease surveillance. Study 1 involved a cross-sectional observational study among the veterinary profession in Australia using an online questionnaire and is reported elsewhere. Study 2, the focus of this manuscript, involved semi-structured qualitative interviews with a cohort of private veterinarians participating in Study 1 for the purpose of obtaining more detailed information on the following key questions:

1. What is the current contribution of private veterinarians to the Australian surveillance system?
2. What is the veterinary professions capacity to assume a more prominent role in surveillance?
3. What is the interest and ability of the veterinary profession in Australia to undertake this surveillance role now and into the future?

## Materials and methods

Human ethics approval to conduct this study was obtained from the Charles Sturt University Human Research Ethics Committee on 31st May 2018 (Protocol number H18096), with data being collected between February and June 2019.

Potential participants were identified from those who expressed an interest in participating in a follow up interview after the completion of an online questionnaire. Given the qualitative nature of the study, the aim was to have a purposive sample of individuals that represented the diversity of the profession in terms of demographics and work type. Purposive sampling in qualitative studies has been shown to demonstrate greater efficiency when compared to random sampling (33). Twenty interviews was considered be sufficient to reach saturation of the information obtained, when additional data gathered does not contribute significantly to the diversity of the results obtained (34). If saturation was not reached with the proposed number of interviews, additional interviews would be conducted.

The following criteria was used for inclusion: must have worked in practice in last 12months ***and*** not working in companion animal practice only, and ***then*** a diverse sample was selected by year of graduation, location of practice and main species serviced (beef cattle, equine, dairy cattle, sheep, goats). From the 120 private veterinarians who indicated interest, 36 were selected, with the assumption that from this group, 20 would consent to interview.

The RAND function in Microsoft Excel (2013) was used to select the first 20 private veterinarians to be contacted. After contact attempts with this group were exhausted the target number of interviews had not been achieved, resulting in a random selection of the next 10 potential participants from the list.

### Development of the semi-structured interview

Overall, the interview investigated current engagement and barriers to and motivations for private veterinarians to contribute to the Australian surveillance system. More specifically, this interview consisted of 18 questions covering five key areas; (1) Veterinary Role, (2) Knowledge of surveillance, (3) Current contribution to the Australian surveillance system, (4) Activities performed that support surveillance outcomes, and (5) Potential future engagement in surveillance. Feedback on the interview questions was obtained from veterinary colleagues of the research team prior to the implementation of the study. The list of questions for the semi-structured interview is presented as Supplementary material.

### Conducting the semi-structured interviews

Prior to commencement of the interview, the participant's responses to the questionnaire were reviewed by the interviewer. The purpose of this was to ensure that the interviewer was familiar with previous responses to assist with the building of rapport and to provide context to the interview responses. All participants received a copy of the Participant Information Statement and Consent form prior to the interview. Where written consent was not received prior to commencement, verbal consent was obtained at the time of the interview and formed part of the interview record. The semi-structured interviews were conducted over the phone, audio recorded. All participants were asked the same key questions, with the opportunity to explore different areas depending upon the participant's responses. Prompts were used where necessary to ensure that the interview covered consistent areas of enquiry. Interviews were between 45 to 60 minin duration and were conducted by two members of the research team (LH and JM).

### Data analysis

The audio records of the interviews were downloaded and transcribed verbatim into a Microsoft Word document using a professional transcription service. Interview data were uploaded to qualitative data analysis software NVivo (QSR International Pty Ltd. Version 12, 2018). Thematic analysis is a widely used method for identifying, analyzing and reporting themes within data (35). Data were thematically analyzed based on the phases outlined by Braun and Clarke (35) and were analyzed at the latent level, whereby underlying ideas, assumptions, and conceptualizations are considered to inform the semantic content of the data. Using this method, data were read with initial ideas noted. In the first stage, responses were coded to questions. Initial codes were further developed and collated into potential themes. Themes were re-read and coding adjusted if required.

## Results

Semi-structured telephone interviews were conducted with 17 private veterinarians, a number which was considered sufficient, given data saturation was achieved. Table 1 provides details of the location and a description of the participant's veterinary practice.

**Table 1:** Practice description and location of private veterinarians participating in surveillance interviews, 2019.

**ID**	**Practice location**	**Practice description**
01	Victoria	Clinical residency – mainly dairy cattle, some beef cattle. Small number of dogs and cats.
02	Victoria	Dairy cattle. Education and training for veterinarians.
03	Victoria	Poultry. In addition to being part owner of a mixed animal practice.
04	Victoria	Mixed animal practice - five veterinarians. Seventy percentage small animal 30% large animal (mainly horses and alpaca, some beef cattle, pigs, chickens, goats and sheep). Mainly hobby farmers.
05	Victoria	Mixed animal practice−15 veterinarians. Ten veterinarians work in production animal (dairy cattle, beef cattle, sheep, alpaca) or combination of production animal and small animal.
06	South Australia	Mixed animal practice, predominantly small animal.
07	Queensland	Consultant veterinarian
08	Queensland	Mixed animal practice - 20 veterinarians. Sheep, cattle.
09	Tasmania	Companion animal- interest in small mammals, exotics, reptiles and wildlife.
10	Tasmania	Mixed animal practice - four veterinarians.
11	Tasmania	Mixed animal practice - nine veterinarians. Dairy cattle and beef cattle.
12	Tasmania	Mixed animal locum.
13	Western Australia	Mixed animal practice - eight veterinarians, 40% livestock (beef cattle, horses, sheep).
14	Western Australia	Equine.
15	New South Wales	Small animal practice - 95% dogs and cats. Five percentage hobby farms (alpaca, sheep, beef cattle).
16	New South Wales	Mainly companion animals. Some pregnancy testing and equine.
17	New South Wales	Mixed animal practice - dairy cattle, beef cattle, sheep, goats, alpacas, pigs.

The following sections present the main research questions of the study and the key themes and subthemes identified within each of the topics included in the interview.

### What is the current contribution of private veterinarians to the Australian surveillance system?

#### Knowledge and opinions on surveillance and biosecurity

##### The activity of surveillance

The discussions on what “springs to mind” when the word surveillance is mentioned and the types of activities that surveillance entailed could be described as being a combination of those who provided a theoretical response, such as descriptions of general and active surveillance, and those whose responses were associated with their own veterinary activities and experiences. A key theme that emerged from the latter responses related to vigilance, often described as occurring at an intuitive level.

*So to me I guess it's probably one of those things that you're subconsciously thinking about it but maybe not necessarily realizing you are doing it every time you step foot out on farm. (17)*

There was a degree of disenchantment expressed related to the level of surveillance occurring and the emphasis placed upon related activities.

*So, I used to think it was probably better done than it actually is in reality, I think. Certainly, in our district there's very little if any surveillance done at all at any time. (08)*

Confidence in the protection of Australia's borders as a result of the historical strong performance of border protection and quarantine services, may lower the veterinarian's perception of risk and decrease the likelihood that an EAD will be included on the list of differential diagnoses.

…*if you're into biosecurity, it's (Australia) an island, it's very good biosecurity because geographically we're so isolated. So, I think – I think that leads to practitioners – a certain amount of – what do I want to say? Slackness because there's so few zoonotic diseases and they're just not as aware as other parts of the world. (14)*

The responsibility that such activities entail was noted in comments such as, “*It is a fear that I'll miss an exotic disease. That's my immediate thought. (07)*”. Given the known increased level of mental health issues experienced within the veterinary profession (31, 36) it is important that any fear inducement in veterinarians around being ill-prepared for the responsibility that government has put on the shoulders of the professionals – implicitly or explicitly is considered in discussions related to increasing the involvement of private veterinarians in surveillance activities.

##### Biosecurity in theory and practice

The term biosecurity, and how it is perceived and used in practice was highlighted, particularly by the more experienced private veterinarians, as one area that had changed over time.

…*probably biosecurity as a term was something that I probably wouldn't haven't even been able to draw out or say but I think the principles of biosecurity are not totally new. It's just we didn't call it that really. (05)*

*I think when I graduated, I don't think we knew what biosecurity was. It was not a word that was used in the veterinary profession or the farming professional probably. Correct me if I am wrong, so it's sort of become a bit of a buzz word I guess probably what 15 years ago or 20 years ago and it's greatly increased from there and it's been pushed by government departments far more than it ever was so there is a much greater awareness of it. (13)*

A disconnect between what was learnt at university and what is experienced at a practical level, was described, with the theoretical knowledge not always resonating until applied in practice.

…*it's obviously a subject you have to take but it's not really – but you don't think of it (in) practical terms, I guess. And once I – but once I started in clinical practice and actually started seeing animals and dairy cattle … you do start thinking about it in terms of real life. (01)*

Whilst for another the opposite was experienced.

*I'd say that we learnt a whole lot more about it than we probably do in practice which is a little bit sad... (15)*

Overall, this group of questions demonstrate that there is a broad range in level of understanding and comfort around biosecurity issues and surveillance activities.

##### Current engagement with and contribution to surveillance

The majority of respondents reported some level of engagement with activities that support the surveillance system (Table 2). There were varying opinions in relation to involvement in government funded surveillance schemes, such as the National Significant Disease Investigation Program, with some respondents speaking positively about their experiences and others expressing some frustration with the amount of paperwork required and the level of remuneration.

*It doesn't pay particularly well – no it doesn't pay well at all let me rephrase that. By the time you talk to the client, go and book it in and go out there and do your post-mortem, come back to the lab, pack all the samples up, send them all off and have a bit of toing and froing with the pathologist as the results trickle out and have a bit of toing with the client as the results come in on an hourly basis it's pretty unrewarding work but I personally see it as fairly vital work. (13)*

Similarly, engagement with other support activities, such as herd health and biosecurity planning, was varied.

**Table 2:** Engagement in surveillance activities and programs of private veterinarians participating in surveillance interviews, 2019.

**ID**	**Participate in Government**	**Encourage herd**	**Assist with biosecurity and**	**Presented biosecurity**
	**funded surveillance**	**health plans**	**other components of quality**	**management information to**
	schemes^*^		**Assurance programs**	**farmers**
01	✓	NA^**^	NA	X
02	✓	✓	✓	✓
03	✓	✓	✓	✓
04	X	X	X	X
05	✓	✓	✓	✓
06	✓	X	X	X
07	X	✓	✓	✓
08	✓	✓	✓	✓
09	✓	NA	NA	NA
10	✓	X	✓	X
11	✓	X	X	✓
12	X	✓	X	X
13	✓	✓	✓	✓
14	X	NA	NA	✓
15	X	NA	✓	X
16	X	NA	NA	✓
17	X	✓	✓	✓

* National Significant Disease Investigation Program (NSDIP) or other government funded surveillance scheme.

** NA, The described activity is not relevant due to the nature of the veterinary work undertaken by the interviewee.

##### Perceived role of private veterinarians

Private veterinarians, in general, saw themselves as playing a key role as part of the surveillance system, particularly due to their disease knowledge, relationships and exposure to clients and their animals, rather than as being ‘responsible' for surveillance *per se*.

*I think that the private vets have an extraordinary capacity to fulfill that role because we're the ones on farm all the time and with the knowledge of diseases and things I mean you know you can argue livestock agents are on farm and lots of other – shearers on farm etc so they can have a role in liaising back with the private vets and things but we're the only ones with the full skills to implement the diagnostics and manage that information appropriately and then control – and then go onto disease control. (13)*

*And certainly, a single episode, a single patient doesn't mean anything, but if we see multiple patients coming in with a similar set of symptoms, then that raises the level of suspicion and we go further. (09)*

The benefit of being “on the ground” and the, at times, serendipitous nature of disease detection was a theme that carried through the discussions. This relates to the importance of relationships and is seen to be a key benefit of having private veterinarians engaged in surveillance activities.

*So, I'm inspecting animal groups and I'm also by the way driving along and looking at animals over the fence. So, I'm driving around the district looking at animals and talking to farmers and they're telling me about what disease they've had, and their neighbors have had and their cousins and their brothers and sisters - as in their farms, farming families. (02)*

The value of being on farm and having the opportunity to observe and provide real time advice emerged, highlighting the importance of relationships and communication. However, it was acknowledged that this type of work required a different approach.

*And people don't do what they're told they should do even if they know they should. It has to be more of a discussion. So, it is a different field of work than traditional clinical work. It requires a different consultation style. (02)*

Similar themes were expressed in relation to biosecurity related support, with prioritization, ongoing implementation and remuneration again highlighted.

…*we have got a price there, but generally, it's the sort of thing that if I'm going out and doing some sort of job on the farm, that generally, sort of comes into discussion and just becomes, sort of part of the visit or the consult fee for that day, sort of thing. (08)*

*But when I'm – even if they don't request, I'll always – if I'm at a property I will always mention biosecurity and just tell people, this would be a good place to allow visitors to park. Don't let them into the shed. Let them just look over this fence. Don't let anyone walks into your paddocks. Things like that. (07)*

In addition to this, a number of those interviewed were currently, or had previously been, involved in active surveillance activities. Examples provided included, Johnes control programs, Bovine Viral Diarrhea (BVD) testing, and the National Transmissible Spongiform Encephalopathies Surveillance Program (NTSESP). There was also involvement in informal active surveillance activities, such as maintaining and interrogating clinic datasets to identify anomalies and trends. Another area mentioned was engaging with social media and facilitating support for animal health related concerns reported through these channels.

*But I also spend a lot of time on social media, and I do – if I see something that's sort of rings concerns or alarm bells, then I'll contact that person and try and organize for them to – if they can't get a vet - to get interested. (07)*

Opportunities for further involvement were suggested, with some uncertainty about how to manage the information collected.

…* when people bring wildlife in, I think that we could have a hand in surveying some of the problems we see, but I don't know of where to give that information to. (04)*

### What is the veterinary professions capacity to assume a more prominent role in surveillance?

#### Current challenges

The challenges facing private veterinarians and their engagement with biosecurity could be divided into three main subthemes – the challenges around the concept of shared responsibility, lack of resources and conflict of interest.

#### Responsibility for surveillance

This theme carried through into the discussions on who was considered to be responsible for surveillance activities. The notion of shared responsibility was evident, with animal owners and managers, private and government veterinarians, industry and government all being seen to contribute to activities that supported to the animal health surveillance system.

*In my mind the responsibility is probably with the government vets as well as vets and their contingency. But I think surveillance and being vigilant is everyone's responsibility including farmers and producers and people that own pets that think wow this a bit weird. (15)*

*So I think it's shared responsibility, but for professional farmers, it's important because that's their – they've got to – everyone's got a big responsibility which should be, in some ways, some of it should be industry funded and government funded because there's so much from our exports that we stand a lot to lose…(10)*

At the highest level, government was seen to be a key player, however issues of devolution of responsibility were further described.

*I think surveillance is a governmental responsibility, but unfortunately, government passes the buck. Whether it be commonwealth, state government or local governments, everybody passes the buck to the private sector for everything. (09)*

*Well, I think the governments so thin on the ground now. And it's also everyone's responsibility because it's going to affect everyone that's got livestock if we don't pick up an early case of an exotic disease or a new and emerging disease. (07)*

Responsibility was also in terms of exposure to negative repercussions should things go wrong.

*Yeah, a lot of people can have a role in it – where does the responsibility lie – is an interesting question like whose backside is on the line? (02)*

The potential for government to shift additional responsibility for surveillance activities was flagged as an issue.

*I'd be worried that if they relied more on private vets, would there be … cuts to government then? I'd be worry about that. And I guess, if you were going to rely more on private vets, then just making sure that they're trained, able to actually to do more. (01)*

#### Lack of resources

The reduction in government funding for extension, government veterinarians and pathology services, was found to have a negative effect on the way in which a number of private veterinarians described biosecurity and surveillance activities, with a level of cynicism evident.

…*that's really down to its bare bones now and there were a lot of extension officers which there are not anymore. (10)*

Concern related to the level of funding and support available was again expressed.

*Putting it out into practitioner's hands at the local level, that's good, but you need someone organizing it. So, I guess that's what I would say about surveillance, you have to have an internal surveillance as a veterinarian and a professional. And then you have an external in surveillance which is regulatory, and it has to be funded, someone's got to pay for it. (14)*

*And the other thing is, straight away they don't want to pay either, so all this talking about surveillance, I suppose I've become a lot more cynical about it all. (06)*

*And then the money dried up so once again it came back to unless it was seen as a disaster on a particular farm, a lot of things probably just died and were put in a hole and put down to snake bite. It sounds cynical but that's probably what it was. (11)*

Encouraging the development of herd health plans, whilst viewed as an important veterinary activity, was not without its challenges. Remuneration for such activities was variable and generally considered to not be commensurate with the time required to provide this service. In addition, a lack of implementation of plans by producers did lead to some questioning the value of spending time and resources on this activity.

*Yeah, and I don't think we were paid well enough to it. I think it was, kind of expected from the client, to be done for free, rather than for a fee, but I do think it was beneficial, because we do have some knowledge for herd health and preventing diseases, and then managing outbreaks when they do happen. (04)*

*I personally love doing herd health and love that sort of work but yeah, it's probably not as valued by the producers or it's hard to sell the value of that and then it's also hard to get the appropriate remuneration for how much work and that that you put into it. (17)*

*I can't imagine too many lawyers – well no offense if you've got any lawyers you know, like just doing things for nothing, like but the expectation. (04)*

*Can be the time and trying to get compensated directly for that. Implementing it, the big concern is all these things can look really marvelous on a bit of paper. If they don't get implemented, then it's a waste of everyone's time basically and that's a bit of my concern with some of them that they've got to be practical. (05)*

#### Conflict of interest

Livestock owners and producers were considered to be vital for the system to operate effectively, however issues such as vested interest and a reluctance to engage private veterinarians who would then be required to report to the government system if a disease issue is uncovered were seen as potential risks. The veterinarian will then be conflicted between servicing his paying client and undertaking required reporting to the government, the client will likely be upset with the veterinarian or may choose to use another veterinarian if the first one reports them as they need to under legislation or animal health policy.

*You can't rely on the individuals to do that – something like this because you're actually asking them to dob themselves in when there's a problem. So, I don't think leaving responsibility with the individuals is going to work because when you get an irresponsible individual the system collapses. (02)*

Private veterinarian involvement in surveillance and the associated responsibility was not without its challenges. The importance of private veterinarians feeling comfortable in admitting to not having all the answers when presented with clinical signs outside what they would expect was expressed and is something that needs to be managed to ensure that appropriate and timely enquiry takes place.

*And I think that has to do with a non-experienced practitioner, well if I refer, I'll lose the client so I can't do that, I can't tell them I don't know. So, I think it is in knowing what you don't know and seeking help. But I think it holds the profession back a lot. (14)*

### What is the interest and ability of the veterinary profession in Australia to undertake this surveillance role now and into the future?

#### Potential future engagement in surveillance

Across all areas of discussion, the overall impression was that there was no clear opposition to private veterinarian's involvement in surveillance related activities. Those interviewed were positive about undertaking additional training if they felt they needed to refresh their knowledge and skills, with a preference for practical training with opportunities to network and socialize rather than attending “*talk fests*”.

A number of suggestions were provided in relation to how to improve animal health surveillance. Providing private veterinarians with the opportunity to “split” their roles between government veterinary activities and private practice was suggested as a way of responding to changing needs.

*I think the veterinary workforce is really shifting toward part-time employment and people do want the best of both worlds. And that doesn't mean that they're working any less hard, but it means that their roles are a little bit different to how they used to be 20 years ago. So, I think if those roles were shifted a little bit then like you say doing LLS [Local Land Services] stuff or surveillance stuff one day a fortnight would be a different way to embrace the same stuff. (15)*

Improved efficiencies in reporting systems, cross information flow and changing the level of confidentiality around positive test results to allow neighbors and communities to be more aware of local risks were other suggested improvements. While these are not areas that private veterinarians can necessarily control, they are important considerations particularly as they have the potential to be barriers to increased engagement.

When posed with a hypothetical situation in which private veterinarians were paid to undertake surveillance as part of a more formal arrangement, a number of themes related to the barriers and benefits emerged. While many were similar to the benefits previously discussed, additional concepts arose, such as bringing different perspectives into the surveillance space along with bridging the gap between private and government veterinarians. Improving relationships and networks across the surveillance space was also highlighted.

*I guess it's probably also a more efficient use of resources. I mean you've only got so many vets in the public sector and that's only so many brains – that's only so many sets of eyes. If you are engaging the private sector that's more brains, more eyes, more ideas, then more ability to get a … of work done as well. (17)*

*Yeah, as much as anything it's a great networking opportunity and I think for a lot of the LLS vets they probably feel quite isolated and exist separate to the normal or commercial vets or whatever you want to call them. (15)*

*One of the issues I don't know if you've covered it yet the people who work in the government and work in disease surveillance can get – and that's all they do become quite one eyed and quite closed in their opinion to how practically farmers work and so there is danger of having people just doing surveillance all the time and part of your question here is about doing a bit of practical work and a bit of surveillance and that would be one of the advantages because it would give people doing the surveillance a bit more knowledge of what – what are the practical drivers on the farm. And so, there is a problem with people doing surveillance all the time that they don't get to see how farms actually function. (02)*

#### Future challenges and potential solutions

Whilst the overall response to future engagement in surveillance activities was positive, this was tempered by a number of barriers or challenges, the majority of which related to lack of resources (e.g., finance, time), producer buy in and conflict of interest, similar to the current challenges previously identified (section Current Challenges). In terms of ways to address these barriers, incentives were discussed. Approaches included direct payment to the private veterinarian / veterinary practice, a retainer and free or heavily subsidized educational opportunities. There was also a suggestion that the incentive needs to be at the farmer level, to counter the difficulties in gaining access to properties.

There was discussion on how to practically implement private veterinarian engagement in surveillance, with finance and workload management again emerging themes.

Whilst a small number of those interviewed saw increased engagement as an additional income stream, in general, private veterinarians expressed a need to be not worse off. The altruistic nature of the veterinary profession was also described along with the need to ensure that this was not taken advantage of Table 3 provides example quotations as evidence of these main themes.

**Table 3:** Example quotations from interviews to support the theme of Future challenges and potential solutions.

***Resourcing - time and financial***	*I hate to say it, but I don't think they can pay me enough. (14)* *No I think we have enough – well certainly busy enough that we couldn't fit much more in without upping the vet numbers and that's another story…(11)* *So whether they can afford to let a vet go and do some Government work for a period of time that might be a challenge to get that system up and running. (16)* *Yeah, I think in a perfect world, people probably would do it for the greater good, but I don't see – yeah, I think that vets commonly do things for the greater good, and absorb the cost, but it does have an impact, particularly when there's drought or when there's other things going on in the community that are impacts on finances. (04)*
***Professional interest***	*… it's not the sort of work I see my career advancing in because you could spend a - it's like being a fire fighter - you can spend a long time sitting around waiting for your fire. I'm out doing things that are helping people now not waiting for something to happen in the future. (02)*
***Access to farms***	*… you've got to get on farm to do surveillance, so how do you get on farm and there are some farmers who like I say they want to diagnose everything themselves. They are happy to put things down to snake bite and they are happy to buy cheap drugs off some unethical vets who don't really care what they are selling, and you can miss it, so the secret is to get on farm. (11)*
***Farmer compliance***	*Time and money and farmer compliance to – well farmer wanting to do it – but there is a lot of farmers that probably need it; most don't necessarily get involved with. (05)*
***Conflict of interest***	*So, there's those issues of on whose behalf you are acting and whose best interest do you have at heart. (02)* *… some of the cons might be that you know in the private sector where we've built up such a good rapport with most of our clients that you have got that massive trust basis there and it may be a bit of a conflict of interest, or appear to be a conflict of interest if you're then working in that sphere as well and potentially having knowledge on both sides of the fence that could affect things. (17)*

## Discussion

Private veterinarians are an integral contributor to an effective animal disease surveillance system. In Australia, it has been shown that areas with the highest surveillance activity intensity align with the locations of private and government veterinarians (17). Private veterinarians are at the forefront for the detection of disease and have further potential to capitalize on existing relationships with producers and other stakeholders to improve surveillance outcomes.

This study has found that private veterinarians are aware of their responsibilities and are engaged in surveillance activities at both formal (e.g. government funded surveillance schemes) and informal, (e.g. observation and education, supporting herd health plan development and aspects of Quality Assurance programs), levels. Considerations related to the efficiencies of the existing surveillance system are important as the frustrations expressed may serve as a barrier to increased future involvement. Whilst there is certainly goodwill with regards to further involvement, the capacity for a more structured role is dependent on considerations such as workload and remuneration in particular. The barriers identified in this study are similar to those reported by Steele et al. (37) who list client-related factors, workplace environment and access to external technical support as key impediments to investigations of atypical disease events. Given the identified shortage of both private and government veterinarians in some areas (38, 39), many of these may be challenging barriers to overcome without a broader review of the veterinary workforce.

To introduce a more structured system of private veterinarian involvement in animal disease surveillance it is essential that remuneration is provided at a level that, at the very least, does not result in financial disadvantage. A recent Australian study investigating the reasons behind veterinarians decisions to leave clinical practice found that employment conditions such as remuneration and working h played a key role (32). Adding pressure to an already stretched workforce is not sustainable and could have unintended negative consequences (31, 40). It is therefore imperative to understand the level at which such work becomes untenable from a wellbeing, workload and financial perspective. The other component of the current research project (Study 1) investigates the monetary value attributed to having one veterinary staff member working 1 day per week in surveillance. This information will be valuable in better understanding the opportunities for increasing private veterinarian involvement in surveillance activities.

Biosecurity, whilst a high priority for government, is generally not afforded the same level of prominence or consistent application by producers on-farm (41, 42). The findings from this study lend support to this, with a number of those interviewed commenting that producers may not see the value in veterinarians providing biosecurity advice as a distinct paid service. The relationship between the private veterinarian and the producer is therefore critical as it is through the provision of other paid services that surveillance and biosecurity related activities are more likely to occur.

It is here that both the strengths and weaknesses of increased involvement of private veterinarians in the surveillance system are exposed. Despite veterinarians being a trusted source of information, many sheep and cattle properties do not engage the services of a private veterinarian on a regular basis (14). In the event of animals displaying signs of illness, many will attempt to resolve animal health issues themselves and only call out a veterinarian to situations where other attempts have failed (5, 6). Given the extensive nature of Australian farming and the very real shortage of rural veterinarians, such actions are not surprising. However, knowing that timely detection is critical for an effective response to a disease event, this delay has the potential for catastrophic outcomes in the event of diseases such as Foot and Mouth Disease (43, 44).

Reflecting on the current biosecurity and surveillance system and any potential improvements would not be complete with considering a systems-based approach to managing this complex system. Focusing on behavior alone without reflecting on the structural and institutional process within which the behaviors are occurring will result in simplistic responses and limited opportunities for real and sustained change (45). This study, while focusing on veterinarians, will contribute to the ongoing discussions about the social, economic and environmental/institutional system that currently drives surveillance and biosecurity behaviors on farm, in veterinary practices and at region, state/territory and national arenas (22, 46).

As a final point, this study presents limitations that need to be considered. Given that participants were selected from those who had previously participating in a survey on this topic, a heightened interest in the area of investigation is likely. Self-selection bias is a criticism that has been directed toward online surveys (47) and while the researchers do not dispute this possibility, the key findings are considered to be relevant across the veterinary profession and show that even amongst an engaged population, there are barriers that need to be addressed. The use of the telephone interviews also warrants consideration. Critics of this method of data collection have highlighted a loss of non-verbal data, contextual data, and the distortion of verbal data as potentially impacting the quality of data received through telephone interview (48). The alternate argument is that the increased efficiencies associated with telephone interviews make this a valuable tool for data collection in certain situations (49). Within the current study, interviewing veterinarians face to face nationally was not feasible due to logistical constraints. Those conducting the interviews were highly experienced and able to easily establish rapport with participants, thereby eliciting candid and considered responses. The veterinary profession in general, is diverse in terms of demographics, practice type, veterinary activities, and location. We therefore must acknowledge that there may be some areas of veterinary practice that were not represented in this study. Despite this, we consider that those interviewed provided a heterogenous range of opinions and experiences on a topic that is relevant both nationally and internationally.

In Australia, there are no studies that have considered the veterinary professions capacity to assume a more prominent role in surveillance that have addressed this from the perspective of the private veterinarian. As such, the current study has contributed to addressing this gap in research. While private veterinarians have the capacity and willingness to get more involved in animal health surveillance this will be dependent on clearly addressing an already heavy workload, adequate renumeration and clarity of the role they play and responsibilities vis-à-vis their government counterparts. Given the previously described common issue of veterinary workforce shortages, coupled with increased global disease threats, this study has allowed us to draw valuable conclusions that are of benefit both within the Australian context and internationally.

## Data availability statement

The original contributions presented in the study are included in the article/Supplementary material, further inquiries can be directed to the corresponding author/s.

## Ethics statement

All participants received a copy of the Participant Information Statement and Consent form prior to the interview. Where written consent was not received prior to commencement, verbal consent was obtained at the time of the interview and formed part of the interview record.

## Author contributions

Study design: MH-J, ED, LH, JM, and YM. Data collection and analysis: LH and JM. Lead manuscript preparation: LH. Contribution to the manuscript: MH-J, ED, LH, JM, YM, and RW. All authors contributed to the article and approved the submitted version.

## Funding

This project is supported by Meat and Livestock Australia, through funding from the Australian Government Department of Agriculture as part of its Rural R&D for Profit programme, and by producer levies from Australian FMD-susceptible livestock (cattle, sheep, goats and pigs) industries and Charles Sturt University, leveraging significant in-kind support from the research partners. The research partners for this project are the Commonwealth Science and Industrial Research Organization (CSIRO), Charles Sturt University, the Bureau of Meteorology and the Australian Department of Agriculture and Water Resources, supported by Animal Health Australia (AHA).

## Conflict of interest

Author ED was employed by Global Veterinary Solutions Pty. Ltd. The remaining authors declare that the research was conducted in the absence of any commercial or financial relationships that could be construed as a potential conflict of interest.

## Publisher's note

All claims expressed in this article are solely those of the authors and do not necessarily represent those of their affiliated organizations, or those of the publisher, the editors and the reviewers. Any product that may be evaluated in this article, or claim that may be made by its manufacturer, is not guaranteed or endorsed by the publisher.
