# Supplementary material for: Australian veterinarians' perspectives on the contribution of the veterinary workforce to the Australian animal health surveillance system
Source: Front Vet Sci. 2022 Aug 18;9:840346. doi: 10.3389/fvets.2022.840346 (PMC9435963; doi:10.3389/fvets.2022.840346)
Supplement: Supplementary file 1 [file Table_1.DOCX]

**The veterinary profession's contribution to animal health surveillance in Australia**

**Phase 2: Vet contribution to surveillance**

**Veterinary Role**

1. Classify the nature of your practice and practice area *(we might know this information already)*
2. How has your knowledge and opinion of surveillance and biosecurity changed over the time since you graduated?

**Knowledge of surveillance**

1. When someone says surveillance to you – what springs to mind?
2. What veterinary activities could surveillance entail?
3. Who is responsible for these activities currently?
4. Do you think the public are aware of this aspect of veterinary science?

**Contribution to surveillance currently**

1. Can you identify activities that you undertake that would contribute to Australia’s surveillance system?
2. Do you participate in NSDIP program? Or otherwise submit samples on a Government funded surveillance scheme?

**Activities that support surveillance outcomes**

1. Do you encourage your farming clients to prepare herd health plans for their livestock? If so what is the pros and cons of you doing this work?
2. Do you assist farmers in meeting the biosecurity and other components of quality assurance programmes? If so what is the pros and cons of you doing this work?
3. Have you ever presented biosecurity management information to a group of farmers before? If so in what setting and what was the experience like?

**Potential future engagement in surveillance**

1. Have you ever wanted to be more involved in surveillance?
2. Have you had ideas about how animal health surveillance could be done better? If so what are they? *(Ask about technology if it is not mentioned)*
3. How could surveillance activities be added into veterinary practice?
4. What are the barriers (if any) that make it difficult for you to increase your engagement with surveillance?
5. If finance is one of the issues, what kind of financial incentive would be required to be involved?
6. What might be the pros and cons if vets were paid to undertake surveillance?
7. If training was offered on these activities – would you allocate staff resources to do this? (If paid or not paid?)

**Other**

1. Are there any other comments that you would like to be considered on this topic?
